# Assessment of the American Flamingo distribution, trends, and important breeding areas

**DOI:** 10.1371/journal.pone.0244117

**Published:** 2020-12-22

**Authors:** Leopoldo Torres-Cristiani, Salima Machkour-M’Rabet, Sophie Calmé, Holger Weissenberger, Griselda Escalona-Segura

**Affiliations:** 1 Departamento de Conservación de la Biodiversidad, El Colegio de la Frontera Sur, Chetumal, Quintana Roo, México; 2 Departamento de Observación y Estudio de la Tierra, la Atmósfera y el Océano (TAO), El Colegio de la Frontera Sur, Chetumal, Quintana Roo, México; 3 Département de Biologie, Université de Sherbrooke, Sherbrooke, Quebec, Canada; 4 Departamento de Conservación de la Biodiversidad, El Colegio de la Frontera Sur, Campeche, México; Sichuan University, CHINA

## Abstract

The American Flamingo, *Phoenicopterus ruber*, is a charismatic bird distributed throughout the Caribbean, North and South America. Its wide distribution, the complexity of international monitoring due to its capacity for long-distance flying, and a focus mostly on local populations, make it difficult to understand the dynamics between sites. Here, we took advantage of the citizen eBird science project to present a global perspective on the distribution of the American Flamingo, and identify the potentially most important countries for breeding. We obtained 16,930 records for the Americas from the 1960s until October 2018, of which 9,283 could be used for our objectives. The eBird database indicated a considerable increase in the total number of records over the last decade (2010s), probably reflecting an increase in tourism facilities, research investment, technological advancement, interest in conservation, and the worldwide availability of eBird. We also observed a range extension in the Gulf of Mexico in the United States and a significant recolonization in the Florida Peninsula. The apparent range extension to the South is more likely to be linked to biases in the data; for example, in any given country the number of records might reflect either reporting efforts or actual numbers. eBird data confirmed that six countries host the main breeding colonies (Bahamas, Bonaire, Cuba, Ecuador, Mexico, and Venezuela). We suggest three additional countries as potential breeding areas for the species (Colombia, Curaçao, Turks and Caicos Islands) for which more field observations are necessary to support this possibility. This global appraisal of the distribution of the American Flamingo using citizen science data provides valuable information for national and international management and conservation programs such as the need to verify the species breeding status in areas where it appears to be expanding its distribution.

## Introduction

The American Flamingo *(Phoenicopterus ruber* L. 1758; Phoenicopteriformes, Phoenicopteridae), also known as the Caribbean Flamingo, is one of the most charismatic birds and a symbol of the conservation of tropical ecosystems [1, 2]. The presence of this emblematic and cultural iconic bird triggers tourism and many economic activities, particularly in countries that host colonies [3]. At the same time, these activities have triggered conservation actions in favor of the species. For instance, many foraging and breeding areas of the American Flamingo have been decreed as Reserve or Sanctuary. Its conservation is of relevance, not only for the species itself but because it acts as an umbrella species, indirectly protecting many other species in its coastal habitat [4]. Currently, the number of American Flamingos is estimated between 260,000 and 330,000 mature individuals and increasing, making it a species of least concern on the IUCN Red List [5]. Notwithstanding its population status, *P*. *ruber* is a habitat specialist of coastal wetlands, which makes it vulnerable to coastal land-use change [6], adverse environmental conditions [2] such as rising sea levels caused by climate change that might reduce breeding success [7], water pollution, and collision with utility lines, among others (references in [8]). American Flamingos live in shallow bodies of saline, brackish or freshwater, from sea level up to 200 m [5, 9], where they forage and reproduce in groups of up to 24,000 individuals [8]. Flamingos move between foraging sites searching for food [10] especially breeding birds that can change of feeding sites seven times more than nonbreeding birds [4]. During the breeding season (mostly from April to August; [8]), flamingos tend to be more gregarious. The main breeding sites recognized include the Bahamas, Yucatan Peninsula (Mexico), Cuba, Haiti, Dominican Republic, Venezuela, and Galapagos Islands [5]. Recently, Frias-Soler et al. [11] also reported Bonaire as an important breeding site, but they did not provide a criterion to determine its importance.

The most recognized distribution of the American Flamingo encompasses the Caribbean Sea (Netherlands Antilles, Bahamas, Yucatan Peninsula, Cuba, Hispaniola), the northern coast of South America, and the Galapagos Islands [2, 4, 5, 8, 12]. Although the Galapagos population was identified as genetically isolated [11], the population structure of the American Flamingo on the east side of the continent is unclear and subject to debate [2, 11]. More recently, several reports indicated the movement of flamingos toward the United States, colonizing areas in Florida [13], Texas, and Louisiana [2].

The literature on the American Flamingo tends to consider local populations only (e.g., [1, 13]), preventing a broader understanding of the movements between different foraging and/or breeding sites. A global perspective is, therefore, necessary to identify important sites for this species, especially since very little information is available for some localities, such as Guyana and Brazil [14, 15]. This information would benefit conservation and management strategies. Here, we propose to take advantage of the huge database provided by the eBird citizen-science project.

For decades, citizen-science projects have allowed people to contribute to scientific endeavors by collecting and recording data, thus enabling scientists to access large data sets otherwise impossible to obtain [16]. One of the most relevant citizen-science project for bird studies is the eBird project. It consists of collecting and sharing information about the distribution and abundance of birds, thanks to volunteers who submit their observations. Furthermore, the eBird project not only increases the quantity of data available but controls for its quality [17]. It is, therefore, an excellent source for many uses (e.g., species distribution, migration, conservation [13, 17, 18]).

In this study, we generate a current and global portrait of the American Flamingo built on 58 years of data available in eBird to answer the following questions: (1) Do eBird records allow the description of the flamingo distribution across countries of observation? (2) Do current observations coincide with the most recognized distribution of the American Flamingo or indicate the need to update its distribution range? (3) Does recent information imply a reassessment of the main sites where American Flamingo reproduction may be taking place?

## Materials and methods

### Ethics statement

Do not apply to this study.

We extracted American Flamingo records from eBird (https://ebird.org/home) on 14 November 2018, covering 1960–2018 (31 October). Across the literature, different terms are used to refer to eBird information; hence, for clarity sake, we define the terms used in this study. We used “record” to refer to a given observation (sighting) registered by a given birder at a given time in a given place; “individuals” to refer to the number of individuals reported per record; “cumulative number of individuals” to refer to the cumulative number of individuals registered in a given place (a country or a region) meaning that this value probably contains repeat counts of the same individuals. The complete checklist contained 16,948 records, but we eliminated all information outside the potential geographic range for the species (18 European records of birds in captivity), leaving a checklist of 16,930 records. We then applied two filters: 1) to keep only one record for a given location on a given day to minimize redundancy, leaving out 6,324 records, and 2) to account only for quantitative assessments (i.e., records providing the number of individuals per record), leaving out 1,323 records. Our final database consisted of 9,283 records. These records allowed us to build maps in ArcGIS 10.2.1, and an animation file using the Time Series Animation tools in ArcGIS 10.2.1. All shapefiles for the map backgrounds were downloaded from http://tapiquen-sig.jimdofree.com [19].

Because there is no definitive agreement among scientists on the population structure of the American Flamingo, we decided to describe trends in the cumulative number of individuals considering a geographical division which included (1) North America, (2) Central America, (3) South America, and (4) the Caribbean. In addition to this regional level, we examined all data at the country or sub-country level (S1 Table for details on countries included). We used two metrics to report the data: (1) total number of records per region (four regions) and per country (32 countries), and (2) cumulative number of individuals observed per region and per country. We compared the data in this study with the most recognized distribution of the American Flamingo to determine their occurrence in areas that were not previously included within this distribution.

To assess the current importance of each country as a potential breeding site, we split the data between a breeding season (mostly from April to August; [8]) and a non-breeding season (September to March). We established a parameter over the last decade (2010–2018), which corresponds to the cumulative number of individuals reported in a given country relative to the total number of individuals reported globally during the nine breeding seasons. We only considered countries with data collected during the breeding season to avoid biases. The value obtained for the Galapagos Islands was set as a reference to identify the most critical potential breeding countries for the American Flamingo. The population of American Flamingo in the Galapagos Islands is a small reproductive population of 500 individuals [11], but an important breeding site for the species due to its isolation [11] from the rest of the Pan-Caribbean population (as defined by [20]).

To get a grasp on the effect of birders’ “effort” on eBird data, we determined the number of record days in eBird for the countries identified as most important for breeding over 1960 to 2018. We then explored the changes in the cumulative number of individuals relative to the total number of records for each country to understand the evolution of eBird data over time, thus coarsely accounting for birders’ effort on the number of individuals recorded.

## Results

### Distribution of the American Flamingo

Detailed information for all eBird records in the four regions is presented in Table 1, and historical data per decade is found in S1 Table. Below, we present information for each of the four regions and for countries.

**Table 1:** Summary of eBird information for the American Flamingo, *Phoenicopterus ruber*, from 1960 to October 2018 for all countries classified into four regions.

Country	Record	Duplicate	Record_clean	Record_qualitative	Record_final	Number of individuals	Year of the first record
	**North America**						
Bermuda	51	0	51	2	49	65	1969
Canada	2	0	2	0	2	81	1978
USA	1,269	580	689	33	656	3,939	1971
Mexico	3,678	1,275	2,403	301	2,102	570,953	1980
Total	5,000	1,855	3,145	336	2,809	575,038	
	**Central America**						
Belize	1	0	1	0	1	1	2007
Honduras	1	0	1	0	1	1	2011
Total	2	0	1	0	2	2	
	**South America**						
Bonaire	2,370	1,307	1,063	100	963	109,249	1984
Brazil	1	0	1	0	1	150	2005
Colombia	661	202	459	54	405	51,519	1979
Curaçao	1,152	413	739	20	719	37,459	1992
Ecuador	1,772	370	1,402	387	1,015	9,771	1973
Guyana	7	2	5	0	5	76	2013
Suriname	37	13	24	6	18	5,105	2011
Venezuela	714	253	461	132	329	1,036,458	1982
Total	6,714	2,560	4,154	699	3,455	1,249,787	
	**Caribbean**						
Anguilla	13	4	9	0	9	13	2015
Aruba	111	17	94	0	94	152	1986
Bahamas	406	166	240	18	222	67,700	1976
Barbados	29	10	19	0	19	19	2004
Cayman Island	91	32	59	1	58	291	2009
Cuba	2,439	1,109	1,330	197	1,133	300,560	1985
Dominican Republic	440	102	338	37	301	9,681	1981
Granada	1	0	1	0	1	1	2017
Guadalupe	1	0	1	0	1	1	2013
Haiti	60	23	37	2	35	1,239	1987
Jamaica	6	1	5	0	5	7	2016
Puerto Rico	912	307	605	8	597	653	1987
Saint Kitts	1	0	1	0	1	1	2002
Saint Martin	4	0	4	0	4	7	2014
Trinidad and Tobago	110	22	88	3	85	2,279	2005
Turks and Caicos Islands	440	94	346	13	333	29,773	1976
Virgin Island	150	22	128	9	119	3,176	1979
Total	5,214	1,909	3,305	288	3,017	415,553	
**TOTAL ALL**	**16,930**	**6,324**	**10,606**	**1,323**	**9,283**	**2,240,388**	

Record: number of records in eBird database; Duplicate: number of duplicate records; Record_clean: number of records after elimination of duplicates; Record_qualitative: number of records that do not report the number of individuals observed; Record_final: total number of records considered for analysis; Number of individuals: cumulative number of individuals considering all records included in Record_final.

#### North America (Map in S1 Fig)

We obtained 5,000 records of which 2,809 could be used for analysis, corresponding to a cumulative 575,000 individuals reported. Canada and Bermuda presented the lowest number of total records and cumulative individuals. The first record for Bermuda was the oldest (from 1969). However, most records were from the 1990s and 2000s, and no observation was reported in eBird in the last decade (S1 Table).

For the United States, we extracted almost 4,000 cumulative individuals (Table 1). The Gulf of Mexico/Florida registered 93% of US records with 588 records and 3,773 cumulative individuals, with a significant increase since the 2000s (S1 Fig and S1 Table). Note that 77% of records from this area belong exclusively to Florida. The other region of the US (East and West Coast; S1 Fig and S1 Table) represented only 7% of records for the country.

Mexico presented the highest number of total records (>2,000) and cumulative number of individuals (>570,000) in North America (Table 1), representing almost all cumulative individuals for North America (99%). Most of records and individuals correspond to the Yucatan Peninsula, where the first flamingo record dates back from 1842 [21], and data including the number of records and individuals began in the 1980s; since then, both continue to increase (S1 Table). Central Mexico presented very few and recent data starting in 2000 (S1 Fig and S1 Table).

#### Central America

This region had only two records, one for Belize and another for Honduras (S1 Table).

#### South America (Map in S2 Fig)

We extracted 6,714 records of which we used 3,455 reporting a cumulative 1,249,787 individuals. Ecuador (Galapagos Islands) and Bonaire presented high numbers of records. However, the country with the highest cumulative number of individuals was by far Venezuela (Table 1). Brazil and Guyana had a low number of total records and cumulative individuals, and those were reported recently (2000s and 2010s, respectively; S1 Table). Suriname reported approximately a cumulative number of 5,000 individuals, with all data recorded very recently (2010s). From the 1970s to 2018, Ecuador provided a large number of records totalling a cumulative 9,700 individuals (Table 1). The total number of records and cumulative individuals increased each decade, and the 2010s contributed to 72% of the cumulative number of individuals (S1 Table). For Curaçao, Colombia, and Bonaire, almost all records dated back from the 2010s with very high cumulative number of individuals (~ 37,000, 51,000, and 109,000 respectively; Table 1 and S1 Table). Finally, Venezuela had a high cumulative number of individuals reported (> 1,000,000) for a relatively low total number of records (329; Table 1). The cumulative number of flamingos in Venezuela represents 46% of all flamingos reported in eBird. Even if the cumulative number of individuals reported since the 1980s was high compared to other South American countries, most corresponded to the 2010s when 95% of the individuals were recorded (S1 Table).

#### Caribbean (Map in S3 Fig)

We obtained 5,214 records of which we used 3,017 reporting a cumulative 415,553 individuals, 72% of which are from Cuba (Table 1). Almost all the islands reported occurrences of the American Flamingo (Table 1). However, on many islands, the cumulative number of individuals was meager (< 20; Anguilla, Barbados, Granada, Guadalupe, Jamaica, Saint Kitts, and Saint Martin) to low (< 1,000; Aruba, Cayman Island, and Puerto Rico). In general, for these islands, observations began recently (2000s/2010s; S1 Table). Some countries (Haiti, Trinidad and Tobago, Dominican Republic, and the Virgin Islands) had a large cumulative number of individuals reported (between 1,000 and 10,000). Three countries had the highest total numbers of records and cumulative individuals: the Turks and Caicos Islands, the Bahamas, and Cuba (~30,000, ~67,000, and ~300,000 cumulative number of individuals respectively), most of which were reported in the 2010s (Table 1 and S1 Table).

### Updated distribution of the American Flamingo

**Fig 1:**
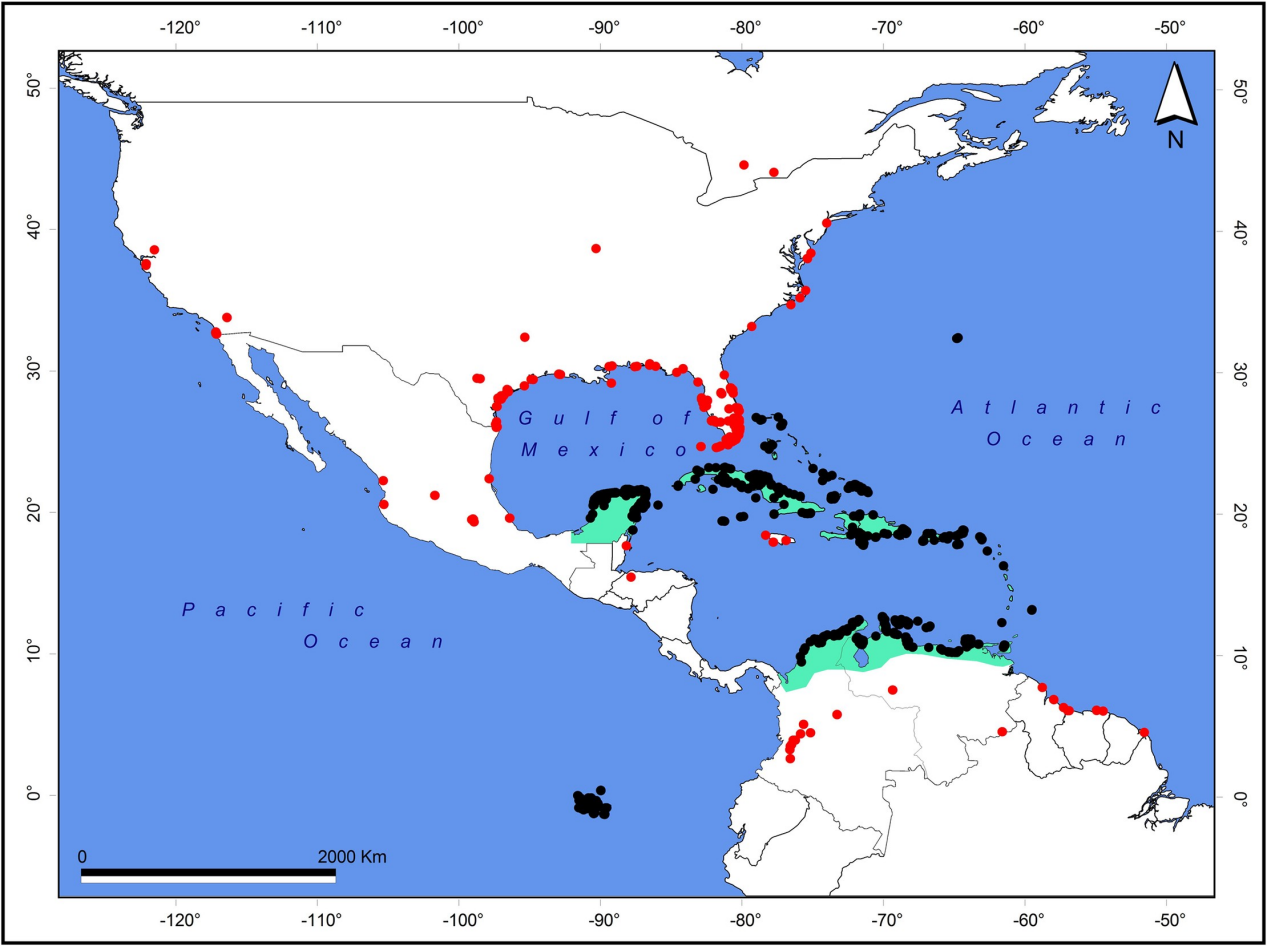
Distribution of the American Flamingo, *Phoenicopterus ruber*, based on eBird records from the 1960s to October 2018. The light blue area represents the most recognized distribution (see Methods); dots represent records inside (black) or outside (red) the most recognized distribution.

When we projected all the eBird records from 1960s to 2010s (N = 9,283) onto a map, we found that most records coincide with the most recognized distribution of the American Flamingo (black dots in Fig 1). However, there were records (red dots in Fig 1) that extended to the North (US Gulf of Mexico/Florida) and to the South (Colombia, Guyana, Surinam, and Brazil).

### American Flamingo breeding sites

During the last decade, the percentage of the cumulative number of individuals observed in Ecuador during the breeding season relative to the cumulative number of individuals observed during that season was 0.6827 (Table 2). Based on this value, we identified nine countries as critical breeding areas for the American Flamingo during the breeding season (in alphabetical order): Bahamas, Bonaire, Colombia, Cuba, Curaçao, Ecuador, Turks and Caicos Islands, Mexico (Yucatan Peninsula), and Venezuela (Table 2 and S2 Table for details per year considered).

**Table 2:** Significance of countries for the American Flamingo, *Phoenicopterus ruber*, during the breeding season (April-August) during the 2010s.

Country	Number of individuals	Percentage of individuals during the breeding season	Number of years	Average of individuals per year	SD	Min	Max
	**North America**							
US- East and West Coast	21	0.0040	2	10.5	NA	1	20
US- Gulf of Mexico/Florida	1,930	0.3650	9	214.4	296.4	4	856
Mexico–Central	10	0.0019	2	5.0	NA	4	6
Mexico–Yucatan Peninsula	117,916	22.3007	9	13,101.8	12,566.4	153	36,083
	**South America**							
Bonaire	21,428	4.0525	9	2,380.9	2,555.3	122	7,782
Colombia	17,900	3.3853	9	1,988.9	2,460.8	20	6,432
Curaçao	11,764	2.2249	8	1,470.5	2,505.0	9	6,740
Ecuador	3,610	0.6827	9	401.1	279.0	57	862
Guyana	40	0.0076	1	40.0	NA	NA	NA
Suriname	295	0.0558	3	98.3	153.9	5	276
Venezuela	243,369	46.0269	9	27,041.0	47,433.0	154	120,211
	**Caribbean**							
Anguilla	7	0.0013	1	7.0	NA	NA	NA
Aruba	142	0.0269	6	23.7	21.1	5	62
Bahamas	11,463	2.1679	6	1,910.5	2,522.3	19	6,788
Barbados	4	0.0008	1	4.0	NA	NA	NA
Cayman Island	9	0.0017	2	4.5	NA	1	8
Cuba	85,007	16.0769	8	10,625.9	21,297.1	36	62,632
Dominican Republic	553	0.1046	9	61.4	84.0	1	261
Granada	1	0.0002	1	1.0	NA	NA	NA
Haiti	435	0.0823	3	145.0	91.5	70	247
Jamaica	1	0.0002	1	1.0	NA	NA	NA
Puerto Rico	172	0.0325	9	19.1	13.3	4	42
Saint Martin	4	0.0008	1	4.0	NA	NA	NA
Trinidad and Tobago	1,726	0.3264	6	287.7	507.1	1	1,273
Turks and Caicos Island	9,688	1.8322	8	1,211.0	1,694.3	37	4,771
Virgin Island	1,269	0.2400	7	181.3	236.2	6	606

Only countries for which data exist in eBird during the breeding season are presented. Number of individuals: cumulative number of individuals; Percentage of individuals during the breeding season: percentage of the cumulative number of individuals in a given country in relation to the total during the breeding season. Countries where percentage > 0.68% (corresponding to Ecuador as reference) are highlighted in light grey; Number of years: number of years with data; Average of individuals per year: determined as Number of individuals / Number of years; SD: standard deviation of the mean; Min: minimum value of the cumulative number of individuals considering years with data; Max: maximum value of the cumulative number of individuals considering years with data; NA: does not apply.

Finally, the changes in the cumulative number of individuals relative to the total number of records for each country (S4 Fig) highlighted contrasting situations. In the nine countries with critical breeding areas for the American Flamingo (S4A Fig), the relative number of individuals tended to increase slightly except for (1) the colony in Ecuador that remained broadly stable, (2) the colony in Cuba that stopped growing in the 2000s, and (3) the colony in the Yucatan Peninsula that appeared to be decreasing since the 1990s. Most other countries (S4B Fig) showed a slight increase in the relative number of individuals since the 2010s. The marked increase in the total number of records and cumulative number of individuals in the last decade (2010s) appears therefore to reflect mainly birders’ reporting effort, as shown on S5 Fig.

## Discussion

This work considered all the information on the American Flamingo available up to date in eBird, a wealth of data for more than 55 years. Looking at regional patterns allowed us to gain a better understanding of its past and current distribution, in particular its likely current range extension. We were also able to pinpoint the location of critical potential breeding sites in some countries, using a simple, more objective criterion. This, in turn, provided a broader view regarding the status of the species that can inform national and international management and conservation programs.

### Distribution of the American Flamingo

To revise the current distribution of the species using eBird data, we developed an overall picture of the situation of the American Flamingo and citizen's efforts to report this iconic bird. Our results demonstrate a bias in the total number of records according to the countries and regions. Countries with high tourist interest and/or high research investment generally reported the greatest total numbers of records and cumulative individuals. The situation of the American Flamingo in these countries is probably well described by the data. For instance, the lack of records in Costa Rica, which is renowned for its ecotourism, particularly ornithological tourism (see [22]), suggests the absence of the American Flamingo in this country. On the other hand, the low total number of records for some countries such as Brazil (Amapa state), Guyana, or Suriname, probably reflect a lack of tourism, the difficult access to flamingo colonies, and/or limited ornithological research, rather than the rarity of the species.

A tendency in many countries was the sharp increase in the total number of records (S1 Animation) in the 2010s and birders’ “effort” indicated by the number of record days in eBird. This may suggest an increase in (1) the worldwide availability of eBird in the 2010s that led to an increase in reported data [17], (2) improved access to technology to share data, particularly the development of smartphone applications that considerably facilitated the submission of data from field [17], (3) the global interest in conservation, (4) research investment, and (5) tourist facilities and especially interest in birdwatching tourism in the whole Caribbean region [3]. Additionally, the increased in the cumulative number of individuals on eBird during the 2010s may also indicate an increase of the American Flamingo global population, as indicated by the Red List assessment which reports the Flamingo population as “increasing” [5]. Our results on changes in the cumulative number of individuals relative to the total number of records for each country suggested a slight increase for most countries, and a decline only in the Yucatan Peninsula since the 1990s. The latter may be caused by decreasing habitat quality, possibly due to natural disturbance [23], which could induce flamingos to move to other sites, as observed in the Galapagos [7]. It might also be explained by the movement of individuals to nearby colonies or feeding areas that showed an increase in the cumulative number of individuals, such as Texas or Florida [2]. Although all other countries (S4B Fig) showed a slight increase in the cumulative number of individuals since the 2010s, very little data is available, so these results need to be interpreted with caution.

In North America, two areas stand out for the American Flamingo: the Yucatan Peninsula in Mexico and the coast of the Gulf of Mexico and Florida. The significance of the Yucatán Peninsula comes as no surprise, as the ecology of the American Flamingo has been studied there for decades (e.g. [4, 8, 24, 25]. Regarding the coasts of the Gulf of Mexico and Florida, eBird data show records there since the 1970s; the last decade (2010s) saw an explosion in numbers, especially in Texas and Louisiana as previously reported (see [13]). These authors suggested that the recent increase of the Florida population probably results from emigration from other Caribbean populations. This resurgence of the Florida flamingo population, eliminated by *ca*. 1900 due to anthropogenic pressure, has generated increased interest in their conservation [13, 20].

In the Caribbean, the three countries that stand out are located in the North: the Bahamas, Cuba, and the Turks and Caicos Islands. In the Bahamas, despite being the national bird [26], the flamingo population was tiny with approximately 100 individuals in the 1950s; however, the increase in the cumulative number of individuals (S1 Table) may suggest an increasing population (S4 and S5 Figs). The group sizes reported in eBird are generally large, with 41% of records reporting flocks of 100 to 1,000 individuals and up to 5,000 individuals in 9% of records. Baltz [26] reported that Flamingos are once again observed in North Andros where they had disappeared by 1950 and that the only current colony is on Great Inagua; in eBird, most records originated from Inagua. In Cuba, which has the highest total number of records and cumulative individuals in eBird since the 1980s, 63% of records report 1–100 flamingos per record; however, some records (5%) reveal flocks of over 1,000 individuals and up to 15,000 individuals. Such large cumulative numbers of individuals in Cuba suggest that some birds are not resident. Indeed, Cuba is an important area for the movement of flamingos during seasonal migration [12]. The Turks and Caicos Islands’ records for date back from the 1970s, but 96% of records did not appear until the 2010s. The status of the species is unclear in these islands: despite evidence of breeding activity and large numbers of individuals [27, 28], Walsh-McGehe et al. [29] were unable to confirm whether flamingos are residents or migrate from areas such as the Bahamas.

Most first records in eBird are from the 2010s in the other Caribbean islands except for Hispaniola and Aruba where the first records date back from the 1980s. On Hispaniola more records are reported in the Dominican Republic than in Haiti, but the population has decreased on the whole island principally due to anthropogenic factors [30]. Flamingo numbers on Hispaniola fluctuate, probably because it receives migrants during the non-breeding season [references in 30]. For Aruba, the flocks reported in eBird have no more than seven flamingos. Luksenburg and Sangster [31] suggested that Aruba’s flamingos may originate from Bonaire or the Los Olivitos colony in Venezuela. In Puerto Rico and the Virgin Islands, although flamingos are reported as vagrants or accidental [32], there is no information published for these locations to the best of our knowledge.

In South America, four countries are noteworthy: Colombia, Venezuela, Bonaire, and Curaçao. For Colombia, Murillo-Pacheco et al. [9] suggested that the recent occurrence of individuals in new areas indicates a possible range extension or the presence of vagrant/introduced individuals. In Venezuela, Espinoza et al. [33] suggested that the American Flamingo population represents 38% of the global population, a very similar proportion to that found in this study. The population of resident American Flamingos appears to have doubled in 20 years, from an estimated 37,110 individuals in 1996 [33] to 75,622 individuals in 2017 [34]. The western coast of Venezuela, specifically the Los Olivitos refuge, is the most crucial area in the country (~69% of eBird flamingo records of Venezuela) [33]. American Flamingos in this area continuously migrate, principally to Bonaire, searching for food [35]. Bonaire is a significant area with reports in eBird spanning several decades, most in the 2010s. The current American Flamingo population of Bonaire is estimated between 1,500 and 7,000 breeding individuals [35]. In nearby Curaçao, the species is considered a regular non-breeding visitor, and breeding attempts of a few hundred individuals were unsuccessful [36]. Although the literature mentions the Jan Thiel Lagoon (a salt pan) as the main location for the American Flamingo [36, 37], eBird reports only 86 records for this area, whereas it reports 293 records in Saliña Sint Michiel (another salt pan).

The literature on the American Flamingo in Guyana, Suriname, and Brazil is scarce. For Guyana, the status is unclear due to the lack of data [14], and recent eBird observations could reflect the opening of this country to eco-tourism [38]. For Suriname, most records are from the Nickerie District (Northern border with Guyana), but Schulz et al. [39] reported the species in the Wia-Wia Nature Reserve, in Central Suriname, over 40 years ago. For Brazil, eBird had only one record with 150 individuals on the north coast of Amapá State in Cabo Orange. This single record is surprising considering that the American Flamingo is a resident species in Brazil [40]; however, it may reflect the isolation and difficult access to the area. This example highlights some of the weaknesses of eBird data, and emphasizes caution during its analysis.

In the Galapagos Islands, the population of the American Flamingo is considered stable, with approximately 500 individuals [41], 45% of which are capable of reproducing [11]. The groups are tiny, with usually no more than 20–30 birds (eBird database, [41]). However, one record in eBird reported a group of 100 individuals observed out of the breeding season (January 2010). Vargas et al. [7] reported that the most important feeding and breeding sites are Quinta Playa and Cementerio Lagoon, both on Isabela Island. eBird data showed only two records for Quinta Playa and none for Cementerio Lagoon, indicating that birds in these sites are probably under-reported. Most eBird records providing a location are for Isabela and Floreana Islands, which have permanent flocks [41], and for which 73% of records date back from the 2010s. There has been a huge increase in land-based tourism in these islands [42], which might explain increasing reports especially around Puerto Villamil on Isabela, while traditional sites could be more difficult to reach.

### Updated distribution of the American Flamingo

The American Flamingo distribution, as reflected by eBird data in this study, may suggest an expansion of its range to (1) the North on the south coast of the United States and in Central Mexico, and (2) to the South in Brazil, Guyana, Suriname, and inland Colombia. The remainder of the records coincide with the species most recognized distribution [2, 4, 5, 8, 12].

The colonization of the North coast of the Gulf of Mexico and the re-colonization of Florida represent the most significant range expansion for the American Flamingo. Although, the number of individuals reported remains modest with a cumulative 3,773 individuals, their increase in this area is dramatic, as just seven individuals were reported in the 1970s. There is evidence that natural dispersion is occurring in the United States with individuals migrating from the Caribbean [13]. This dispersion is consistent with the findings of other studies that demonstrate a northward range expansion for different taxa, possibly related to global warming [43, 44]. These studies suggest that climate change may affect and modify the distribution of the American Flamingo, which warrants further modelling of the potential geographical range.

In Mexico, flamingos were reported in different states (e.g., Mexico City, Jalisco, Tamaulipas, Nayarit, Veracruz, and Guanajuato), but the very low total number of records and cumulative individuals suggested that these individuals were introduced or vagrant. In Mexico, flamingos are uncommon out of their distribution area (the Yucatan Peninsula). However, some locations such as Laguna Chumbeño in the Biosphere Reserve Marismas Nacionales in Nayarit have small population descendent from individuals that have escaped from captivity [10]. Therefore, we can consider that eBird reports in Central Mexico do not represent an expansion of the range of distribution of the American Flamingo.

In Colombia, Murillo-Pacheco et al. [9] suggested the possibility of range expansion for the Orinoco region. However, the eBird data had no information for this region. Historically, the American Flamingo distribution occurred on the Caribbean coast of Colombia [5, 9], as shown by the majority of eBird records coming from coastal Departments (e.g., La Guajira, Magdalena). As in Mexico, inland records of flamingos could be exotic or introduced [9] and these probably do not equate to a range extension.

The southernmost regions where the American Flamingo occurs are Suriname, Guyana, and northeast Brazil [5]. The scarcity of information on flamingos in both Suriname and Guyana is probably due to limited tourism and research in the area. In Brazil, the status of the American Flamingo is unclear; it is reported in the Amapá State where it reproduces [40]. The bird list of Brazil considers the American Flamingo a resident species, but this is unconfirmed [45]. In summary, it seems that information in these countries is more a reflection of the presence of eBird as an easy and readily available means of reporting rather than a valid range expansion.

### American Flamingo breeding sites

Previous literature recognized six countries with important breeding sites for the American Flamingo: Mexico (Yucatan Peninsula), Bonaire, Ecuador, Venezuela, Bahamas, and Cuba [5, 11]. Whitfield et al. [20] omitted Ecuador because they considered only the “Pan-Caribbean population”. Using recent eBird data (2010–2018) for the breeding season, and setting the Galapagos as the reference point, we identified nine countries that are potentially important breeding areas for the species. Six of these countries coincide with those previously mentioned. At the same time, an additional three were included: the Turks and Caicos Islands, Colombia, and Curaçao. The inclusion of these countries was based on our analysis of eBird data and is therefore not confirmed. However, this result should inform efforts to assess the status of the colonies in those countries.

The inclusion of the Turks and Caicos Islands should come as no surprise. When estimating the historical nesting range of the American Flamingo in the Caribbean, Allen [46] already mentioned this British territory. Norton and Clarke [27] found two abandoned nesting colonies on North Caicos with 6,000 to 7,000 mounds, and the presence of young flamingos in the Turks and Caicos Islands strongly suggests breeding in this country [29]. Contrary to the situation in many areas of the Caribbean, where wetlands are disappearing under anthropogenic pressures, wetlands in the Turks and Caicos Islands remain effectively unaltered by human activities [28]. Furthermore, these authors noted that the ornithological interest in the Turks and Caicos Islands remained low in the 1990s, and eBird records for the breeding season did not commence until 2010, which may explain the lack of information.

The situation of Colombia as a potential breeding area is far less clear. There are records of the American Flamingo nesting in the department of La Guajira during the first half of the XX Century, and recent observations suggest the possibility of reproduction [15]. Nevertheless, according to these authors, individuals move to Venezuela (“Ciénaga de los Olivitos” protected area) to reproduce. Indeed, no nesting areas were reported in the 2000s despite efforts to find active nests or mounds [47]. One hypothesis is that flamingos show high fidelity to their birth site [48], and therefore would most probably return to bordering Venezuela to reproduce. However, although eBird has reported the American Flamingo in Colombia since the 1990s, the first information during the breeding season appeared in 2010 with a significant increase in the cumulative number of individuals in 2016 (> 4,000). This data may suggest that flamingos find suitable nesting areas in Colombia, stressing the need to increase efforts to confirm its reproduction status in that country.

For Curaçao, recent eBird data may suggest a small incipient breeding population. Though considered a non-breeding visitor, several breeding attempts were documented [36], and eBird reported two recent events related to breeding. The first, in 2012, corresponded to territorial defense behavior in a group of 3 individuals in Saliñas Jan Kok; the second, in 2018, corresponding to the C code (meaning courtship, display, or copulation) in a group of 120 individuals in Jan Thiel Lagoon. Almost half of records during the breeding season counted less than 20 individuals and did not exceed 200 individuals [49]. Groups of less than ten pairs are unlikely to nest, because flamingos need social stimulation to develop breeding behavior [20]; although, as Lazell [50] found in the Virgin Islands, this is not impossible. Consequently, Curaçao is a potential breeding area, but more research is necessary to confirm it.

### Perspectives

The American Flamingo is a charismatic bird, but knowledge related to its populations and movements remains surprisingly patchy despite its importance. Citizen science projects such as eBird allowed us to obtain valuable information regarding where and when the species is located and how much certain sites are crucial. Nevertheless, a complete understanding of the movements of individuals will only be possible using techniques such as capture-mark-recapture, satellite telemetry, and stable isotopes, among others [13]. Though flamingos are reported in many countries, very little literature is available for most countries. This underlines the necessity to develop more scientific programs that include information sharing and cooperation between countries, to firmly establish the status of the American Flamingo and gain a better understanding of its population dynamics. In the interim, eBird data allows us to pinpoint areas that may require more attention for their conservation, in addition to areas where the species is expanding. Of course, a more detailed analysis of eBird data for each country combined with information on the population dynamics of the American Flamingo is warranted. However, it is beyond the scope of this study. Finally, we suggest a re-evaluation of its status, especially for countries hosting important breeding colonies.

Our results appear to support the current view that the status of the American Flamingo is improving generally [5], in line with the significant efforts that have been made to conserve its populations. In 1978, the Flamingo Specialist Group (FSG; http://www.flamingo-sg.org/), today part of the IUCN Species Survival Commission, was created. This group is composed of flamingo specialists who study, monitor, manage, and conserve the populations of six species of flamingos [15]. At the same time, many countries where the American Flamingo inhabits increased efforts to gather information about the species and to ensure the protection of its habitat. In Mexico, for instance, the scientific interest in flamingos began in the 1950s in the Yucatan Peninsula, and by the 1970s the Mexican government had launched a monitoring program. In 1979, two federal reserves were created in the region to protect flamingo habitat (Ría Lagartos and Ría Celestún), and in the 1980s research efforts turned to habitat use, feeding ecology and behavior, as well as the effects of ecotourism on the species (references in [4]). In Cuba, the population of flamingos was declining due to habitat degradation and hunting, prompting the Cuban government to create, in 1978, the “National Company for the Protection of the Flora and Fauna” to develop a protection plan for the American Flamingo that would allow its recovery [51]. The Bahamas developed specific programs for the American Flamingo, mainly to identify critical habitat and assess the breeding status of the species [26]); Bonaire decreed wetland reserves protecting important areas for the species [52]; Colombia started to legally protect flamingos from hunting in 1971 and created national and regional reserves to protect its habitat [15]; and Venezuela decreed most of Los Olivitos, an important area for the flamingo, a wildlife refuge in 1986 [53]. These great efforts in the Caribbean region have been rewarded so far; however, they must continue due to the vulnerability of flamingos to natural and human disturbances, especially climate change.

## Supporting information

S1 Fig Occurrence in North America of the American Flamingo, *Phoenicopterus ruber*, based on eBird records from the 1960s to October 2018. For the United States of America, we considered two areas: coast of the Gulf of Mexico and Florida, and East and West Coasts. For Mexico, we considered two areas: Central Mexico and Yucatan Peninsula.
(TIF)

S2 Fig Occurrence in South America of the American Flamingo, *Phoenicopterus ruber*, based on eBird records from the 1960s to October 2018. (TIF)

S3 Fig Occurrence in the Caribbean of the American Flamingo, *Phoenicopterus ruber*, based on eBird records from the 1960s to October 2018. (TIF)

S4 Fig Index relating the cumulative number of individuals to the total number of records per decade and per country. (A) Most important countries for breeding, (B) All other countries.
(TIF)

S5 Fig Total number of days with eBird record, indicative of the effort by birders, for the most important countries for breeding over 58 years. (TIF)

S1 Table Summary of the eBird records for the American Flamingo, *Phoenicopterus ruber*, presented by decade and for each country classified into the four regions. The 2010s runs until 31 October 2018. Rec: total number of records; N: cumulative number of individuals. The value 0 means no data in eBird database.
(TIF)

S2 Table Cumulative number of individuals of the American Flamingo, *Phoenicopterus ruber*, for the breeding season over the 2010s decade, only for countries which present data in eBird. Countries highlighted in grey are the areas potentially important during the breeding season (see methods and Table 2 for details).
(TIF)

S1 Animation Animated map of the eBird records of the American Flamingo, *Phoenicopterus ruber*, from 1960 through 31 October 2018. (AVI)
