# Supplementary figures and images for: Assessment of the American Flamingo distribution, trends, and important breeding areas
Source: PLoS One. 2020 Dec 22;15(12):e0244117. doi: 10.1371/journal.pone.0244117 (PMC7755198; doi:10.1371/journal.pone.0244117)

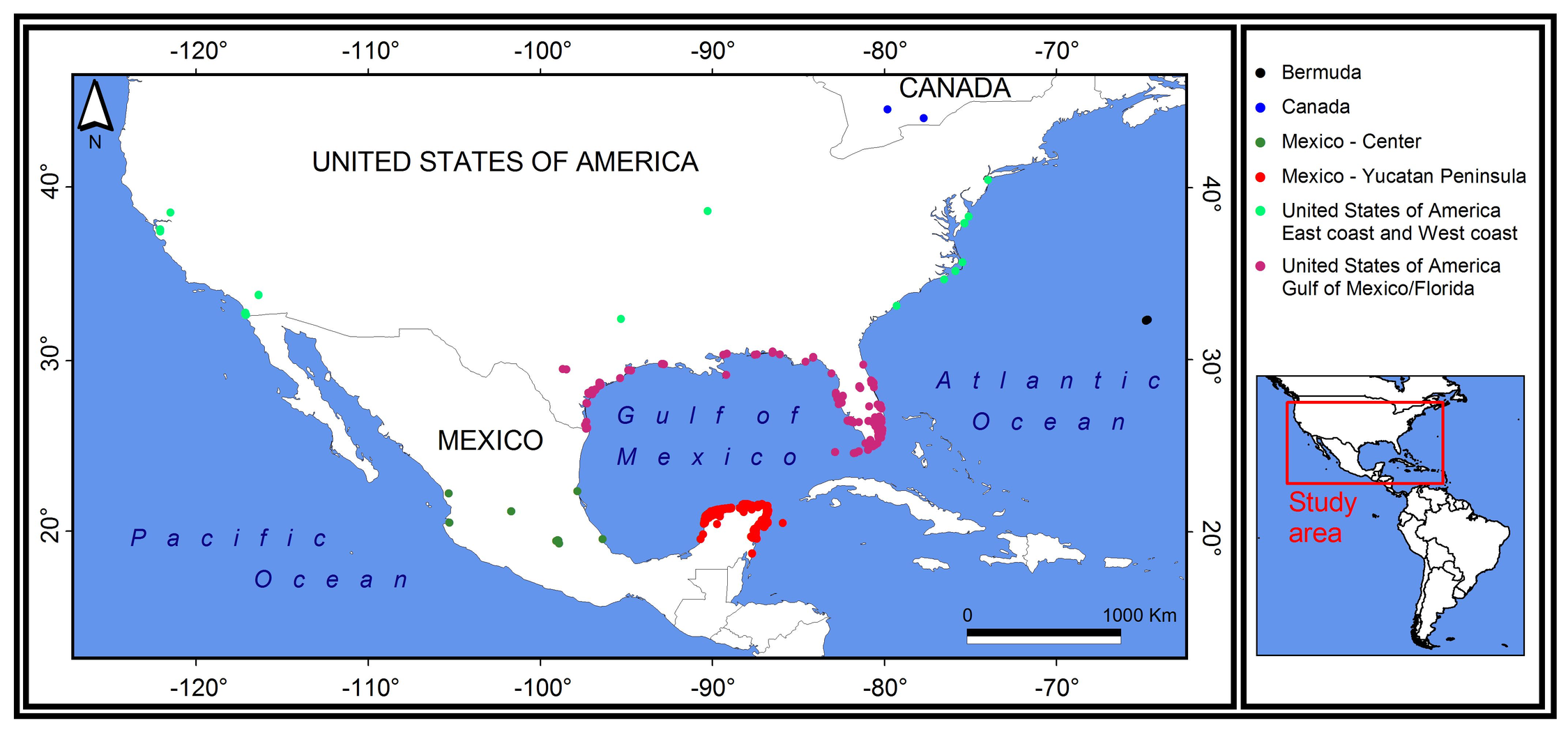

Supplement: S1 Fig — For the United States of America, we considered two areas: coast of the Gulf of Mexico and Florida, and East and West Coasts. For Mexico, we considered two areas: Central Mexico and Yucatan Peninsula. (TIF) [file pone.0244117.s001.tif]

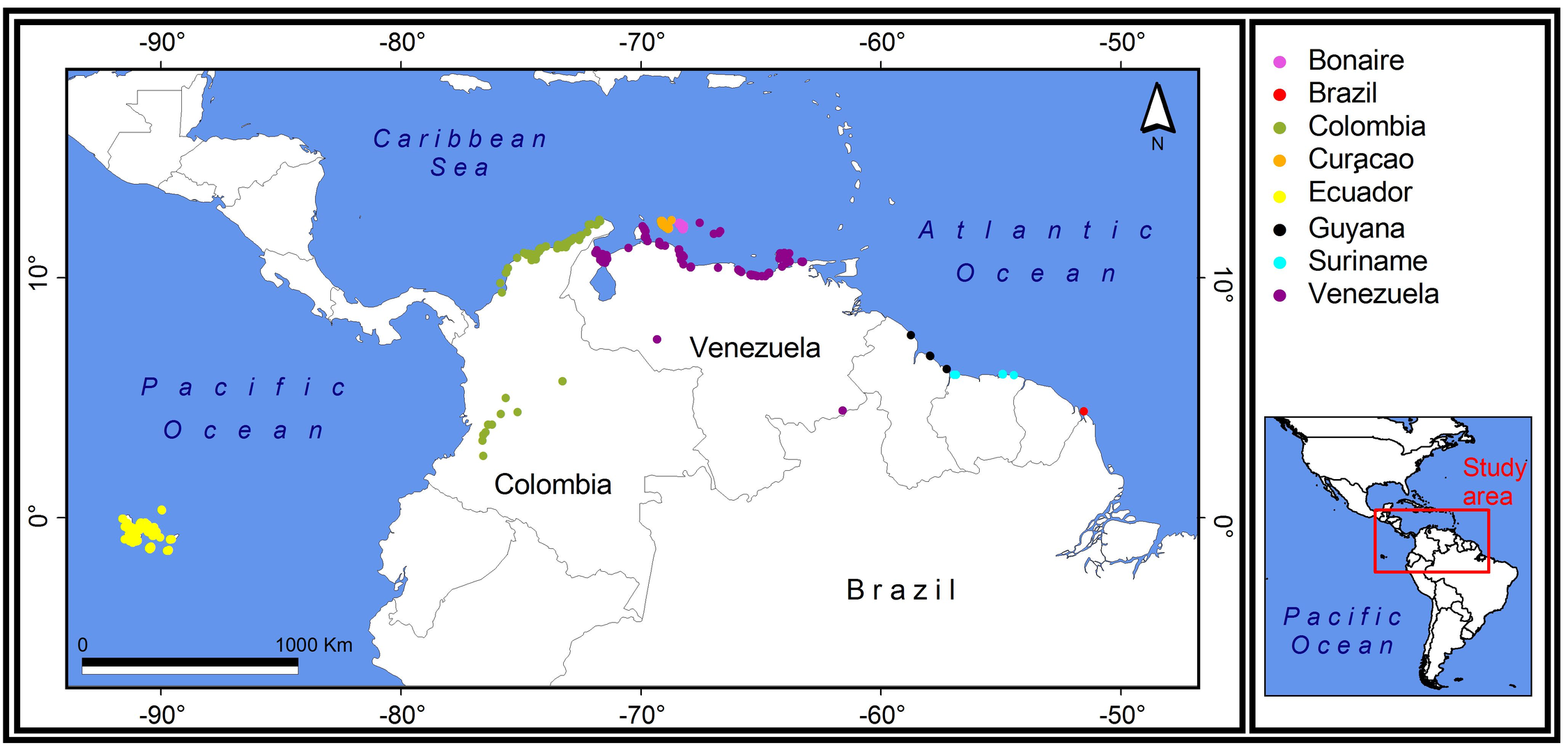

Supplement: S2 Fig — (TIF) [file pone.0244117.s002.tif]

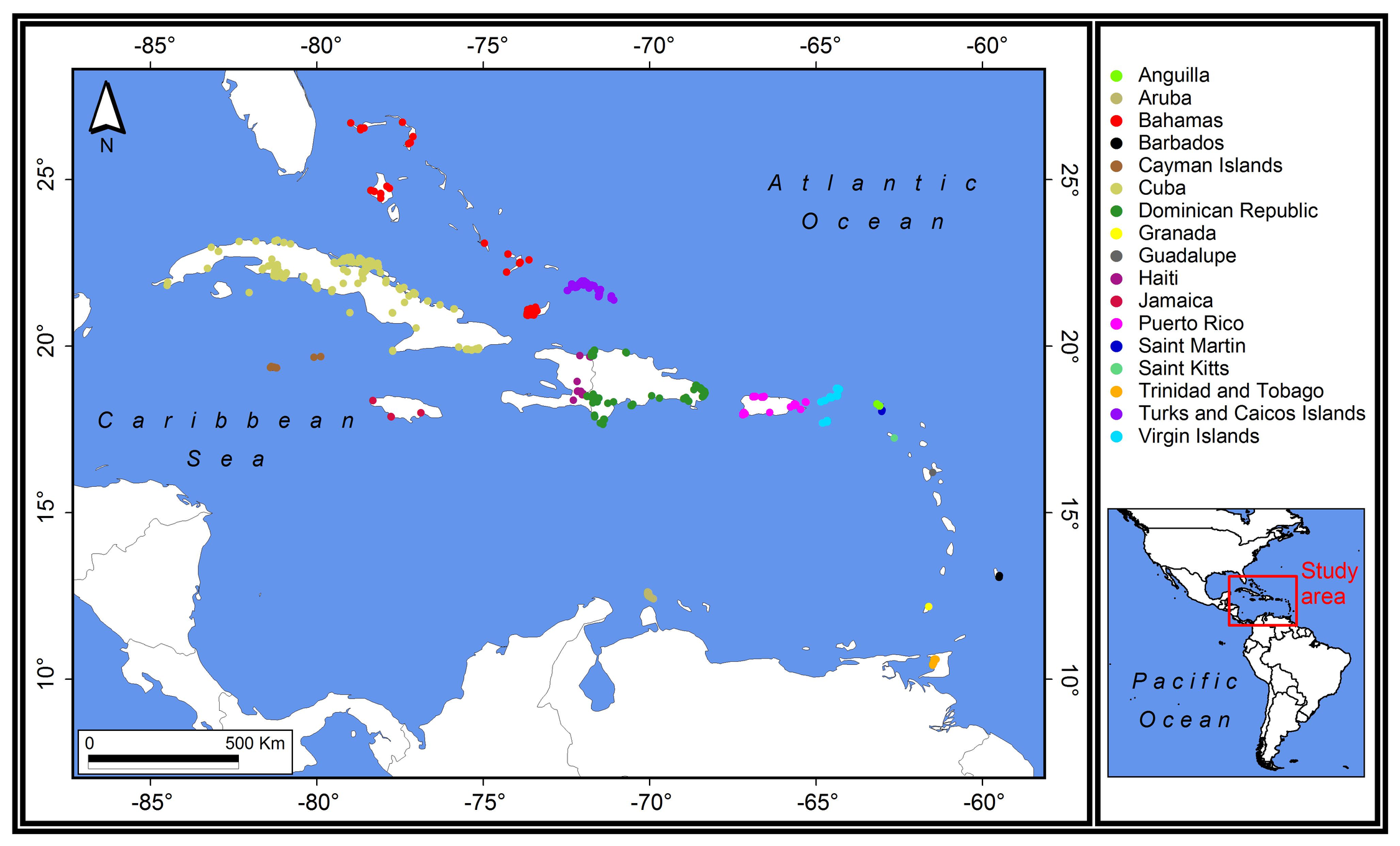

Supplement: S3 Fig — (TIF) [file pone.0244117.s003.tif]

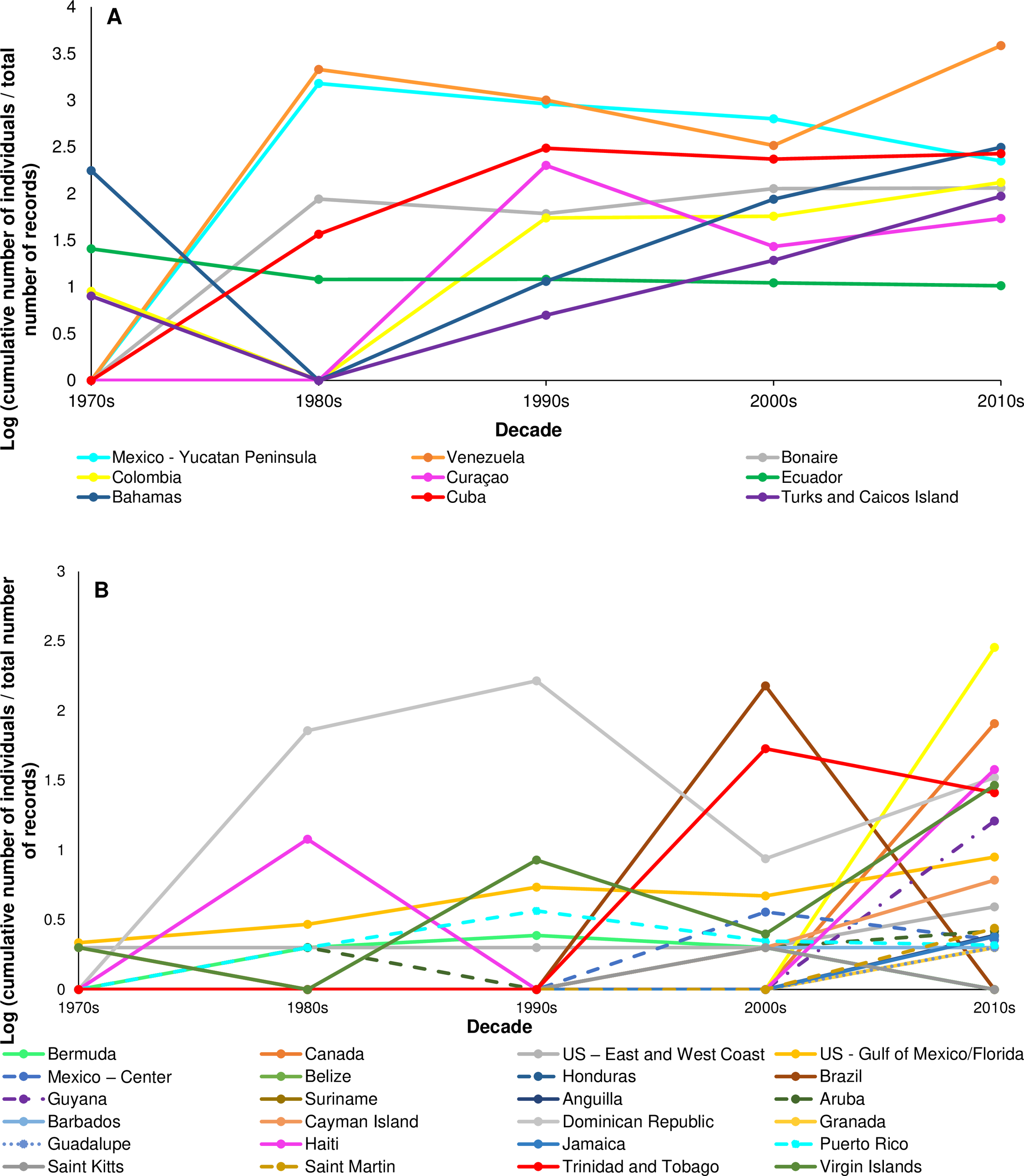

Supplement: S4 Fig — (A) Most important countries for breeding, (B) All other countries. (TIF) [file pone.0244117.s004.tif]

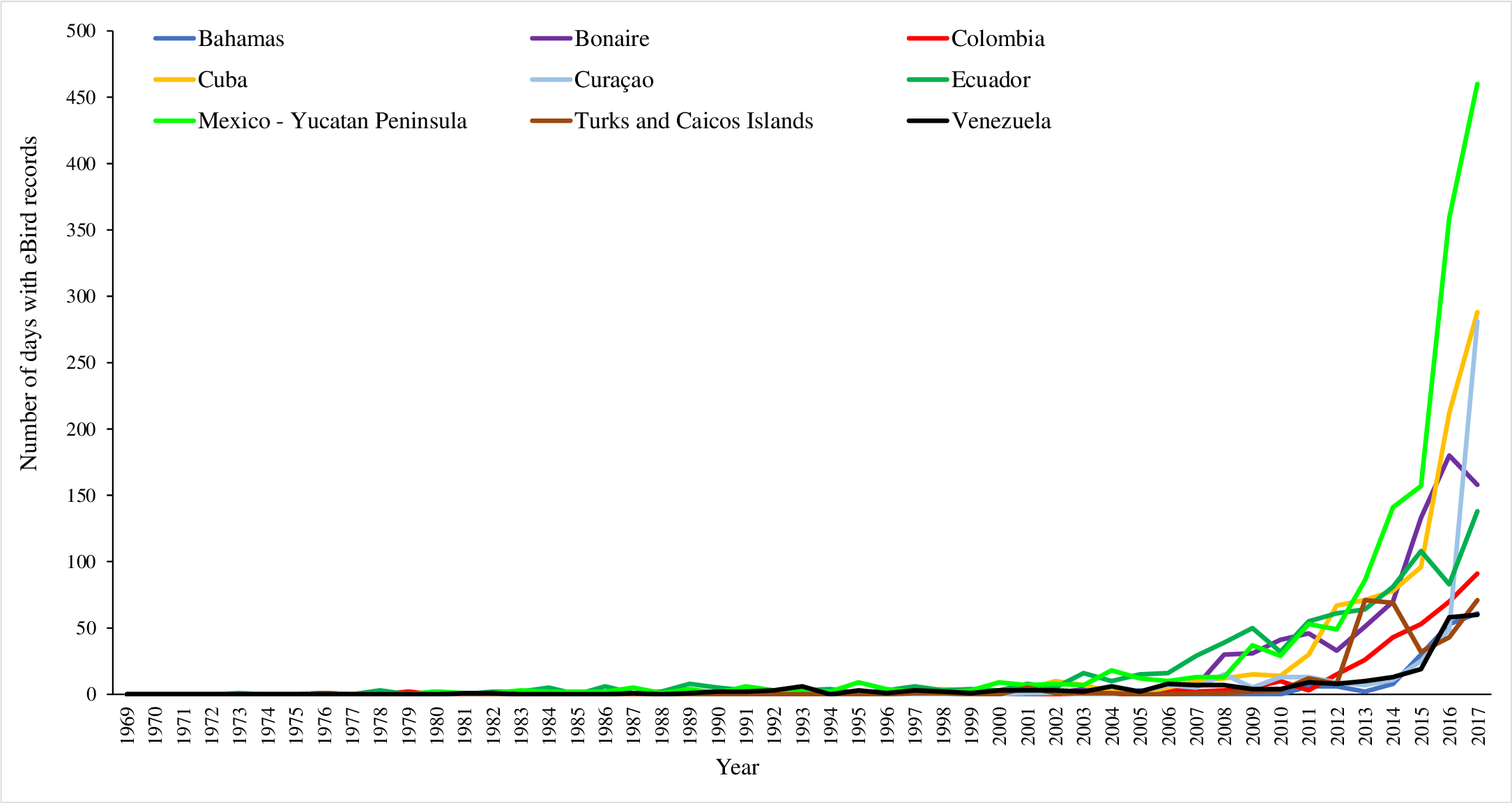

Supplement: S5 Fig — (TIF) [file pone.0244117.s005.tif]

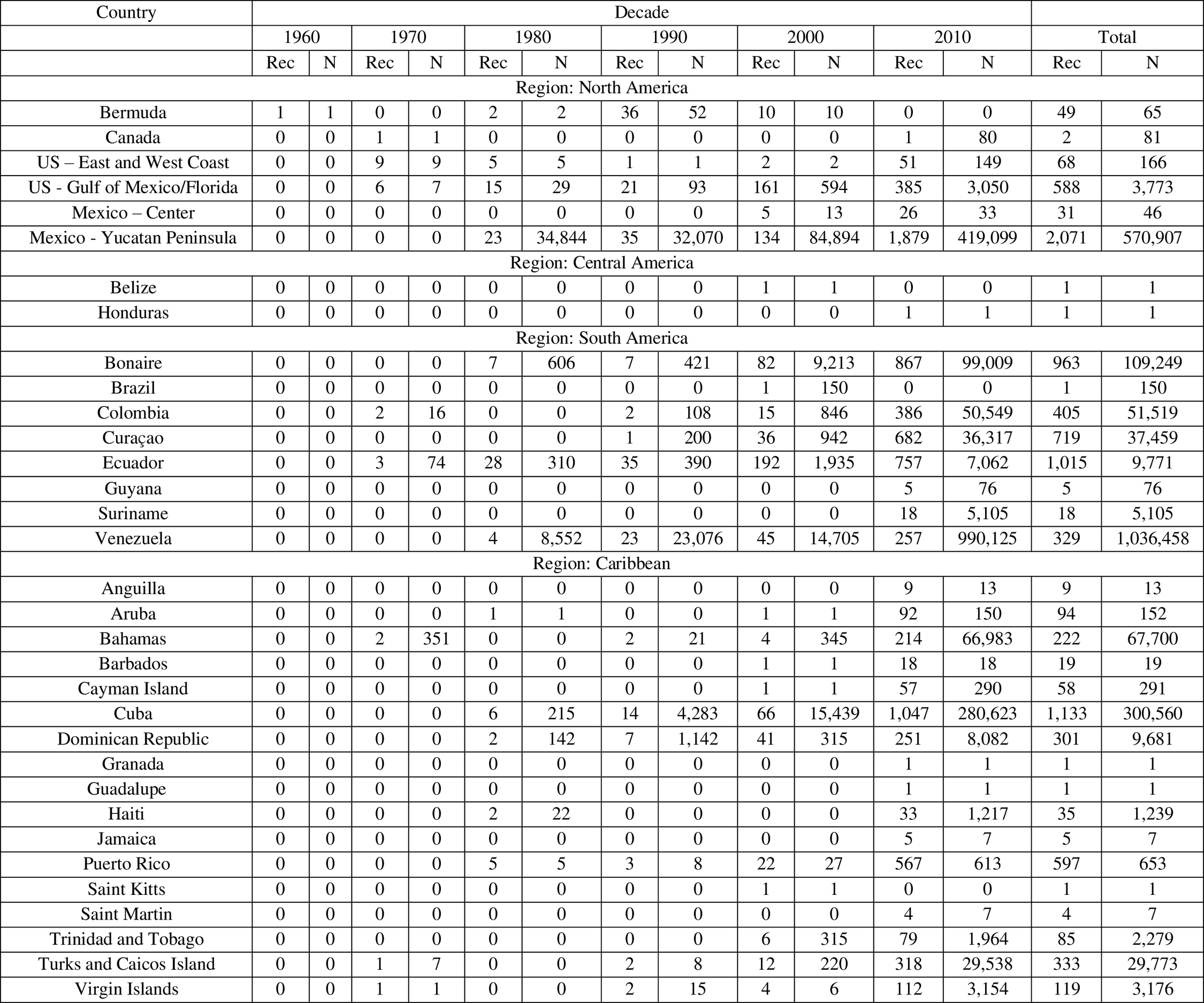

Supplement: S1 Table — The 2010s runs until 31 October 2018. Rec: total number of records; N: cumulative number of individuals. The value 0 means no data in eBird database. (TIF) [file pone.0244117.s006.tif]

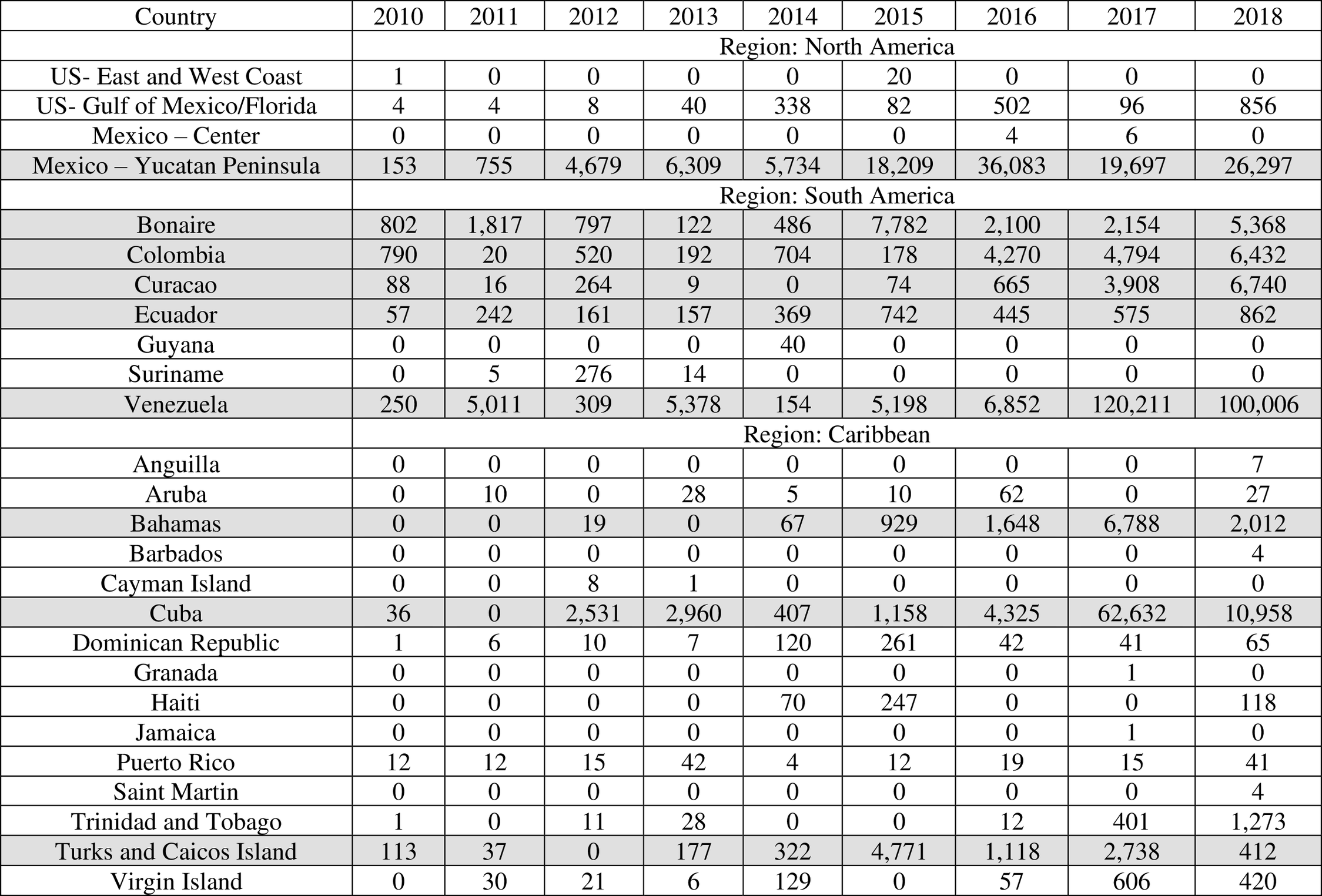

Supplement: S2 Table — Countries highlighted in grey are the areas potentially important during the breeding season (see methods and Table 2 for details). (TIF) [file pone.0244117.s007.tif]
